# The influence of Chile’s food labeling and advertising law and other factors on dietary and physical activity behavior of elementary students in a peripheral region: a qualitative study

**DOI:** 10.1186/s40795-023-00671-7

**Published:** 2023-01-11

**Authors:** Franziska Pfister, Claudia Pozas

**Affiliations:** School of Health Professions, Bern University of Applied Sciences, Murtenstrasse 10, 3008 Bern, Switzerland

**Keywords:** Childhood obesity, Nutrition environment, School, Food policy, Chile

## Abstract

**Background:**

In 2016, Chile implemented the Food Labeling and Advertising Law to fight childhood obesity through front-of-package food labelling, marketing restrictions and school activities and programs. Nevertheless, little is known on its influence on key stakeholders in vulnerable peripheral regions of the country. This study aimed at identifying important influencing factors including the Food Labeling and Advertising Law on dietary habits and physical activity patterns of second graders in Chile, as perceived by school representatives and the children themselves.

**Methods:**

Semi-structured interviews with four school directors and 17 groups of three second graders, informal interviews with 9 key teachers and 4 food services staff complemented with documented observations were carried out in four primary schools of the Chilean city of Punta Arenas, in the Magallanes Punta Arenas region. The different sources allowed for triangulation of results.

**Results:**

FLAL seems to have a negligible influence on young children’s diet and physical activity in the study region. Barriers are children’s deeply rooted dietary habits, excessive screen-time, the parents’ lacking problem awareness, limited time for parenting practices and bad role modeling. Environmental barriers are overloaded schools due to insufficient coordination between governmental entities, lacking funds for sound teacher training and unsafe neighborhoods limiting children’s play.

**Conclusions:**

Policy interventions aimed at reducing childhood obesity need to include and empower schools, families and local communities as active partners and consider their framework conditions for greater influence on dietary habits and physical activity.

## Introduction

In Chile, almost 29% of children between 4 and 14 years of age were reported to be overweight and 24% obese in 2019 [1]. National data suggest a steep increase in obesity prevalence between the first and second grade (6–8 years), the lowest socio-economic groups being more strongly affected [1, 2].

As in other regions of the world, the high overweight and obesity rates are a consequence of rapid nutritional transition and increasingly sedentary behavior in the Chilean population [3]. In 2007, expenditure in processed foods amounted to 57% of total food expenditure [4]. On average, Chileans consume 30 kg of ultra-processed food products and 170 l of sugar-sweetened beverages (SSB) per year, which makes them the leading consumers of SSB worldwide [5, 6]. Children and adolescents consume too much sugar, fat and salt and do not achieve guideline recommendations for physical activity [7, 8].

To prevent childhood overweight and obesity, the Chilean Government ratified the Food Labeling and Advertising Law (FLAL) (Law 20 606/2012), taking effect in January 2016. It covers four aspects:

1. Foods and drinks high in calories, sugars, sodium, and saturated fat must be marked with a front-of-package label (FoPL). The limits for these critical nutrients were lowered in three stages. Thus, while in 2016 a food product had to carry the warning sign for ‘high sugar content’ when it contained more than 22.5 g of sugar per 100 g, this limit dropped to 10 g in the third stage in 2019.
2. Products with FoPL are subject to restricted advertising and marketing. Targeting children younger than 14 years is prohibited.
3. Products high in the above-mentioned critical nutrients may not be sold in schools, be they packaged or not.
4. Schools must provide nutritional educational and promote physical activity [9, 10].

First evaluations suggest a substantial reduction in sales of high sugar/sodium/saturated fat-products at school kiosks in the capital, and a significant reduction in SSB consumption in urban areas [11, 12]. One year after FLAL implementation, mothers in the Chilean capital stated that they perceived an important shift towards healthier dietary habits [13]. Nevertheless, little is known about its implementation and influence in peripheral regions like the Southern region of Magallanes, where prevalence of childhood obesity is among the highest in the country [1]. One of the key settings of FLAL implementation is schools. Therefore, it is crucial to understand how the Law is implemented in schools, and which influencing factors have been identified by school representatives for its successful implementation. Further key stakeholders in the school setting are students. Little is known about young Chilean children’s perception of the measures, despite them being potential key informants on their lifestyle choices and the factors that influence these (e.g. [14, 15]). To address this knowledge gap, the current study identified important influencing factors including the FLAL on dietary habits and physical activity patterns of second graders in the Chilean region of Magallanes, as perceived by school directors, staff and the children themselves. This knowledge may contribute to law amendments as well as the design of current and future policy measures in Chile and other countries.

## Methods

### Study design

We chose a qualitative description research design in order to develop an understanding of the factors influencing dietary habits and physical activity patterns in our target group. A special focus was placed on FLAL as perceived by the students and the school representatives, as the implementers of the Law [16]. CP collected all data in October 2018 in the city of Punta Arenas, Magallanes region, while visiting the four primary schools willing to participate. She had spent most of her life in this region working in the public health sector before carrying out this study.

Four semi-structured interviews with school directors, 17 group interviews with second graders (7–8 years old), documented observations at the four schools, and nine informal interviews with school staff important for the implementation of FLAL like sports teachers and food services employees were carried out. All data was collected directly at the participating schools. Interviews were held in Spanish. As children feel more comfortable talking with peers [17], the interviews with them took place in a group setting of three. The different sources of information (interviews with school directors and students, participant observation, informal interviews with school staff) allowed for method and data source triangulation, leading to a more comprehensive understanding of the phenomenon under investigation [18, 19].

### Schools’ and participants’ recruitment

The 14 primary schools in Punta Arenas classed as Health Promoting Educational Establishments were contacted through the Health Promotion Unit’s local representative, to ask whether they were willing to participate in the study. Schools belonging to this unit are committed to the promotion of a healthy lifestyle and participate in a corresponding governmental program. They are evaluated on a regular basis by the Ministry of Health [20]. The four schools which authorized the researcher to be present during regular school activities were included in this study. By approving the research, school directors agreed to participate in the personal interviews. Participating second graders were chosen randomly from the group which had been given parental authorization, with a minimum of six participants per school. The interview partners for the informal interviews were selected through convenience sampling at each school.

### Data collection

In October 2018, CP visited each school for three entire days, during which the interviews with directors and second graders were carried out. The three children of each interviewed group belonged to the same class and were therefore familiar with each other. The conversation with the children took place in a separate, empty room at school to ensure privacy and diminish distractions. As recommended in the literature [21, 22], the interviewer used appropriate language and talked to the children in a familiar manner, to stimulate participation. As CP stayed at each school for three days and was present during regular school activities, the problem of children feeling anxiety due to the presence of unknown adults was minimized. Informal interviews were held with actors crucial to FLAL implementation (e.g. physical education teachers, cafeteria staff, kiosk manager). The perspective of those working with the children daily was thus captured. The questions were asked in relation to the topics from the interviews with the children and answers obtained thereof. All interviews lasted between 15 and 35 min and were voice-recorded. Interviews were complemented with documented observation of aspects relevant to the research question (e.g. snacks brought to school by the children, left-overs after lunch, physical activity during the breaks and during gym class; activities led by school staff). To record this data, notes and photos were taken.

### Instruments

The children’s interview guide was based on the ecological model of Birch and Ventura [23]. This model comprises three layers of factors influencing a child’s weight, namely child behavior, parenting and parent behavior and community and demographic characteristics. Accordingly, the interview guide included short, specific, and one-dimensional questions on the following four topics:

1. Nutrition: Dietary habits, personal food preferences and basic knowledge on healthy nutrition and the FoPL.
2. Physical activity: Way of commuting to school, favorite activity during breaks, attitude towards physical education classes, participation in new school-based exercise programs, leisure time activities.
3. Parents: Caretaking (presence, cooking, family activities) and physical activity.
4. Community-specific factors: Access to and consumption of fruit and vegetables, neighborhood security.

Due to the reduced time-span children that age (6–8 years) are able to concentrate, a duration of 30 min was aimed for and proved to be sufficient for answering the leading questions.

The interview guide for school directors mainly focused on the school environment. There were six leading questions regarding childhood obesity: changes observed in children’s dietary and physical activity habits after FLAL implementation, unchanged habits, strengths, and weaknesses regarding FLAL implementation in the respective school, parent support and proposed changes for a more effective healthy lifestyle promotion.

### Pre-test

The children’s interview guide was pre-tested at the Latin American School in Bern, Switzerland, and adapted thereafter thoroughly. Both interview guides were checked for face validity by local representatives of the Chilean Health Promotion Unit in Punta Arenas and pre-tested again at the first school in Chile. As no changes had to be made thereafter, this data was included in the results.

### Data analysis

All audio recordings were transcribed ad verbatim. Notes and pictures of observations were stored in a separate file. The data was analyzed according to conventional, structural qualitative content analysis: [24–27]. In a first step, CP read the interviews several times to become familiarized with the data. In a second step, she developed in-vivo codes based on a data-driven approach. Then sub-categories and categories were formed. Coding was done with ATLAS.ti software (Windows, version 8). In total, 37 codes were established, grouped into fourteen sub-categories and four categories in an iterative way. For this publication, quotations were translated into English.

### Ethical approval

The study was carried out in accordance with the Helsinki Declaration for biomedical research. The Magallanes region’s Health Ministry (Secretaría Regional de Salud de Magallanes y Antártica Chilena) gave its consent to this study (date of approval: 19.01.2018). Furthermore, permission to carry out the research on campus was given by the school directors. Parents of the interviewed children and interviewees gave their written consent to participating in the study. Children gave their verbal consent. As no health-related data was collected, the approval of the Bern Cantonal Ethics Committee was not necessary according to the Swiss national regulations (Federal Act of 30 September 2011 on Research involving Human Beings, Switzerland).

## Results

Four primary schools participated in this study. Two of the schools are public, two are private with subsidies from the municipal government (Table 1). Three of them offer free school meals for children of disadvantaged families. All schools are attended mostly by middle class students, have high obesity rates and the government label for healthy lifestyle promotion.

**Table 1:** Characteristics of recruited schools from the city of Punta Arenas and number of interviews carried out per school

Schools	Characteristics				Interviews			
	**Funding**	**Childhood Obesity Rate** **at School**^a^	**Socio-economic Status of Students**^b^	**Students’ Sex**	**Children (# of groups)**	**Directors** **(# of persons)**	**Additional staff** **(# of persons)**	**Cafeteria staff and kiosk managers (# of persons)**
School 1	Public	≥ 35%	Middle/low	Mixed	5	1	6	1
School 2	Public	≥ 35%	Middle	Mixed	4	1	0	2
School 3	Private/subsidized	≥ 30%	Middle/low	Girls only	6	1	2	1
School 4	Private/subsidized	≥ 25%	Middle/(high)	Mixed	2	1	1	0
**Total Interviews**					**17**	**4**	**9**	**4**

Sources: ^a^[28]; ^b^[29]

A total of 21 interviews were carried out: 17 with groups of children from the different schools and one with the director of each school. Further, 13 informal interviews were held with teachers, cafeteria staff (school meals) and kiosk managers (Table 1).

To improve readability, the results were grouped into four categories: school environment, family environment, children’s awareness and behavior, and community environment.

### School environment

The four schools had implemented several strategies to improve child nutrition: Information leaflets about healthy child nutrition had been distributed to parents, and playful pedagogical tools rewarded children for bringing a healthy snack. Observations revealed, however, that many children consumed energy-dense snacks like cookies and juices. Others brought the healthy snack only for getting the reward. In an informal interview an interviewee stated:

*Sometimes they bring the fruit for “Peraman” [“Pear man”; pedagogical tool] only and then throw it away (Assistant, School 4, informal interview)*

Three of the four school kiosks had closed after a drop in sales, as their offer had been reduced by the FLAL to healthy foods. The fourth kiosk continued to operate and offered salads as a snack. However, an informal interview revealed that foods banned by the FLAL from kiosks were still available at schools, as some students had started to secretly sell junk food.

*The older girls sometimes sell candy at school because there is no kiosk anymore. I don’t criticize them, but I think they are very intelligent and entrepreneurial (Teacher, School 3, informal interview)*

The composition of school meals had changed due to the FLAL. Calories had been reduced, for instance, by decreasing the fat content of flavored milk and the sugar content of cooked fruit, and increasing the offer of vegetables. Yet, the informal interviews with cafeteria staff revealed that many children left them on the trays due to the different taste, as they did with the vegetables. Moreover, sweets and unhealthy snacks continued to be consumed for celebrations. For the festivity of Teacher’s Day for instance, CP observed that mothers brought cakes, pastries, potato chips, juices and soft drinks for breakfast. They explained in an informal interview that, after the Law had taken effect, they had organized celebrations with healthy foods, but children had not eaten them.

The schools had also implemented strategies to increase the physical activity of their students by making breaks less sedentary, for example. The fitness equipment on the playgrounds however was used mainly by children of the first four grades. No teacher-led organized activity could be observed during the breaks and children did not use the games painted on the floor.

*Especially from the fifth grade upwards, […] the girls sit in the breaks […] The use of the cell phone is a priority tool for them and this makes them indifferent to some of the activities we organize. (School director, School 3)*

Observations and informal interviews revealed that during physical education class, the children were sitting around or playing games of low physical activity. Children stated that the classes were boring because the activities were repetitive.

*I don’t find them entertaining at all, they are repeated all the time. […] And when they tell us we’re going to do tests, we always play tag. We could play other games. In first grade we played at climbing ropes but now we don’t anymore (Children group 4, School 2)*

Obese children only performed low-impact activities. Teachers stated in the informal interviews that they lacked the necessary tools to support them and feared that they might present some health problem in class. Some parents exempted their obese children from physical education.

*Obese children do not participate in physical education, it is complicated because they get tired. As a teacher one does not know how much effort they can make without having a health problem. (Physical education teacher, School 1, informal interview)*

To fight childhood obesity, the schools offered a range of extracurricular physical activities, either as afterschool activity for children or on Saturday afternoons for the whole family. However, few attended, and often not those with weight problems.

*On Saturdays the physical education teacher has been organizing afternoon games with fun dance for the whole family, […] those who go are few, […] and those who go are people who do not have the problem of obesity and those who have it do not come. (School director, School 3)*

In general, teachers’ motivation for the topic was often limited, mainly due to three reasons: Firstly, many of them felt that parents were not concerned about childhood obesity, and that they gave all responsibility for its management to the school.

*I feel that parents don’t care until they see them [the children] sick, with a prediabetes or something like that. (School director, School 4)*

Secondly, many programs and activities regarding childhood obesity were being offered by various government institutions without coordination. Schools were often overrun with various similar activities, without follow-up or reporting of results and thus felt a lack of coordination, efficiency and even of continuity from the government.

*I think it would be good to have better coordination […] Suddenly there is a program that comes from the CESFAM [Family Health Centers, first health contact point], one from the SEREMI [regional department of the Chilean Ministry of Health], another from the Municipality and it is like they all aiming to the same thing with activities that are a little different, but among them there is no coordination (School director, School 2)*

### Home environment

Most of the parents worked and came home much later than their children, many of them single parents. After school children were left in the care of grandmothers, siblings or alone. Some had to take care of younger brothers or sisters. Many children therefore prepared their meals alone based on the available ingredients, food preferences and cooking ability.

*When I get home, I make my own noodles […] I love them (Children group 3, School 3)*

Even if the parents cooked, they usually prepared fast and convenience foods, as time to prepare more elaborate meals was lacking.

*[…] now there are many more mothers who work, there are single mothers who must work until 8 or 9 pm; then there is no time to make a space to cook. One understands that the poor lady must arrive tired (School director, School 2)*

Many of the families had limited access to healthy foods and considered these expensive. Parents thus gave their children mainly cookies and juices as snacks, although children knew that these are not healthy.

*[…] I prefer to eat [snacks] without sugar but sometimes as my mom doesn’t have that much money and these [cookies with sugar] are sometimes cheap, and she buys me these (Children group 6, School 3)*

Children usually went to school by car or school bus. Many children said they disliked walking, amongst other things because of the bad weather. Daily physical activity was therefore lacking. Children moreover felt that parents did not motivate or support them to participate in sport or physical activity after school.

*No, my mom doesn’t take me to soccer anymore because she always has important things to do (Children group 1, School 1)*

On the other hand, children reported having access to electronic devices such as cell phones and television, regardless of their family’s socioeconomic status. Almost all children owned a mobile phone, even in schools in the most vulnerable areas. The use of electronic devices was rarely controlled by parents or caregivers. Children did not have any supervision regarding electronic device use or bedtime.

*Does anyone tell you to stop watching TV or cell phone? No, I turn it off when I get hungry (Children group 4, School 1)*

*No, I stay up all night watching videos (Children group 6, School 3)*

Another problem proved to be parents’ perception of their children’s nutritional status. Often, they did not seem to recognize overweight or obesity in their offspring. Some of them felt offended if their child was considered overweight or obese. Others believed the excess weight to be temporary and assumed their child would reach normal weight in adolescence due to growing.

*[…] they do not see the problem, and if they see that they are chubby they think "it is healthy and it will grow [...] they are significantly overweight, but as they have not presented any disease yet, it is not of concern (School director, School 4)*

Even when parents were aware of their children’s unhealthy nutritional status, many did not take them to the health center for monitoring. Children thus did not receive the necessary attention, potentially worsening their weight problem over the years.

### Children’s awareness and behavior

Children knew that foods with warning labels were unhealthy. They identified "junk foods", usually relating to fast foods such as French fries, as well as healthy foods such as fruits and vegetables.

*I: What are these [black labels on cookie packaging]? K: They’re high in calories, sugar. When they have that, they’re junk [food]. (Children group 2, School 3)*

Nevertheless, children described that it was hard to resist junk food. The same proved to be true regarding drinks: Although children knew that consuming water was healthy and essential for their growth, they chose what they liked best and were used to from home: Sugar-sweetened beverages.

*What I like to drink most is soft drinks (Children group 3, School 3)*

*They don’t drink much water. Here there is no culture of drinking water (School director, School 4)*

Yet, children mentioned that they liked and consumed fruits. Their parents offered them fruit and reinforced that they were good for their health.

*My dad tells me “what kind of food do you want to take”, and I choose an orange or sometimes a banana (Children group 4, School 2)*

Most of the children ate only the vegetables lettuce, tomatoes and carrots. Many others were not even known by name, had never been tried before but were disliked anyway. When asked about the dishes they liked, children usually named those consumed at home like rice, meat, noodles, sausages and mashed potatoes. At school they tended to refuse to eat foods not familiar to them.

*[I: What is the most common thing you eat at home?] K1*+*K2: Mashed potatoes with sausage. K1: Mashed potatoes is what I like best. K3: Hamburger, eggs, sausages. Today at my grandmother’s house there is mashed potatoes and sausages too. (Children group 4, School 2)*

*They don’t eat the vegetables, they eat only what they know and eat at home, rice, sausage potatoes (Cafeteria staff, School 1, informal interview)*

Almost all children stated that access to unhealthy foods at home was easy. Although parents often hid and consumed them in secret, children knew about this and helped themselves without their caretakers noticing.

*K1: They [parents] buy a big bag of snacks and say that the four of us are going to eat them, and always at night I go to bed and the three of them stay there eating them. I know where they are so I go to get some “ramitas” [a sort of snacks like chips] and eat them in my room.*

*(Children group 2, School 3)*

As mentioned before, many of the children spent time alone after school and decided how to use their free time. They chose to be online or sit in front of the television, with unlimited access to children’s programs, movies, games and videos.

*K1: I’m not so much about going out and playing, I’m more about cell phones. K2: I’m more of a TV guy (Children group 4, School 1)*

At the same time, children stated that they often got bored with these activities and felt trapped.

*I’m only on my cell phone because I’m bored and don’t know what to do (Children group 1, School 3)*

### Community environment

Playgrounds and squares however were not always in good condition and considered unsafe by parents and children due to the presence of marginalized people, aggressive stray dogs and heavy traffic on the surrounding streets. As a result, parents did not let their children go there alone and children themselves were scared to go.

*They [parents] won’t let me go alone. […] one day there was a mattress lying on the floor and there was a drunkard sleeping and I ran away with my sister (Children group 2, School 1)*

*There’s a dog that foams at the mouth and he bit my friend one day and that’s why they won’t let me go outside […] (Children group 6, School 3)*

Security issues were also one of the reasons children did not participate in extracurricular activities after school.

The bad weather was another important reason why children did not engage in outdoor activities in their spare time. In some cases, caregivers wanted to prevent them from getting sick.

*One time we went out for a run [in physical education class] and a boy’s mother complained that it was too cold. Yes, and now we’re not going. Now we can’t go outside anymore (Children group 2, School 1)*

## Discussion

To our knowledge this is the first study that explored how school representatives and children perceive the implementation and effects of the Chilean Food Labeling and Advertisement Law (FLAL), as well as the factors influencing its effect. Our results indicate that the Law has little influence on the nutrition and activity behavior of primary school children and their families in Magallanes. Children know about healthy and unhealthy food, but this knowledge contrasts with their dietary habits and personal preferences. Although they know the meaning of the new FoPL, they do not adapt their food choices. The parents’ lacking problem awareness regarding childhood obesity, unhealthy nutrition, lacking physical activity and excessive screen time counteract the public health objectives set with the FLAL implementation. Parents’ options appear to be severely limited by unfavorable working conditions, however. Schools on the other hand are overloaded with the different demands placed on them and with the lack of coordination between authorities. The findings of this study confirm that without changes in the family, the community and the working environment, it will be difficult to decrease childhood obesity in peripheral regions in Chile.

### School environment

The importance of schools in preventing childhood obesity has been acknowledged by education stakeholders all over the world [30–33]. School programs have been successful in preventing childhood obesity in different contexts, especially if they include interventions combining diet and physical activity [34–37]. The FLAL mandates Chilean schools to promote nutrition education and physical activity. The current study found that schools in peripheral regions are making some efforts to improve their students’ dietary and physical activity habits. The focus seems to lie on written nutrition information, a reward system for healthy snacks and the provision of fitness equipment. However, teachers lack adequate pedagogical tools to teach about healthy lifestyle in an effective and sustainable way. Kain et al. found that expert support was crucial for a successful school-based intervention in Chile [38]. The lack of trained staff being able to facilitate fun and engaging physical activities for students with differing fitness levels was also identified as a barrier to physical activity at public schools in Australia [39]. This matches our findings. Nutrition-specific training, on the other hand, was shown to enable classroom teachers to play a key role in preventing overweight in their students [40]. Furthermore, for effective and sustainable school programs, curricula need to be revised thoroughly. In a meta-synthesis of qualitative studies, school stakeholders considered it important to integrate healthy eating messages across the whole curriculum [32]. In their recent research, Correa et al. showed how the FLAL-promoting role of Chilean schools was lost when they were closed during the pandemic, especially in more vulnerable population groups [41]. A systematic review and meta-analysis showed that nutrition education programs being delivered across two or more traditional primary school subjects and using experiential learning approaches proved to have the biggest impact on primary school children’s nutritional knowledge and behavior [42].

Previous studies in the school setting have shown that children expect their teachers and other school staff to be role models for a healthy lifestyle [30, 32, 33, 43]. Interestingly, this was mentioned neither by students nor the school staff in the present study. A possible explanation thereof was that as many of the staff were overweight themselves, they may have been aware of the inconsistent message they would have been sending to their students.

As a further contradiction, the current study found that on the one hand children receive nutritional education, while on the other hand unhealthy food is consumed on special occasions and for celebrations. The latter is consistent with research carried out in California, which reported that a significant amount of unhealthy foods and drinks are brought to schools for classroom rewards, celebrations and fundraising [44]. This practice might create a strong association between special events and unhealthy foods in children. Furthermore, Clarke et al. showed that children tend to associate foods permitted by and provided by schools with healthy nutrition [32], which might exacerbate the contradiction mentioned above.

School representatives also perceived difficulties engaging parents in obesity prevention efforts. Insufficient parental involvement in healthy lifestyle interventions in schools has also been described elsewhere, in Chile [38] and abroad [32]. At the same time, education stakeholders in other studies highlight the importance of educating and empowering parents to make better lifestyle choices [32, 39]. Clarke et al. suggest that a conflict exists between schools and parents about whose responsibility it is to ensure a healthy lifestyle for children [32]. Our findings support this position from the point of view of school directors and staff.

The Chilean FLAL prohibits the sale of products high in critical nutrients in schools. Unhealthy school meals were perceived as a major obstacle to healthy nutrition by education stakeholders in different studies [32, 39]. Our findings indicate, however, that while the involved schools have started to offer healthier school lunches as a response to the Law, the students seem to resist the adaptations. Indeed, in a qualitative systematic review, children’s preference for unhealthy food and their rejection of new, healthier foods at school was identified as a barrier in the prevention of childhood obesity [32].

School kiosks are a further focus of FLAL, as they are not allowed to sell products with warning labels anymore. As a result, there was a substantial reduction in the availability of foods high in calories, sugar and saturated fat in school kiosks in the capital [11]. Our study suggests, however, that the Law may also have undesired side-effects, namely the closing of school kiosks. This affects the livelihoods of kiosk operators, while unhealthy snacks continue to be consumed at schools because the regulation of unhealthy foods does not include foods brought from home and bought elsewhere. In the capital, schools have started to restrict snacks brought from home according to the number of FoPL [13]. Nevertheless, approaches based on restrictions might not promote a true change in awareness and lead to compensatory overconsumption of unhealthy foods outside of school. A study from the UK found that parents had diverging opinions about whether school policies should comprise rules for healthy snacks [32]. In Portuguese schools a healthy snack policy was shown to encourage the consumption of fruits, however [45]. Interestingly, in the present study the only kiosk that still operated was selling healthy foods successfully, showing that a healthy offer may influence students’ snack choice. Further research is necessary to identify promotors for such positive examples, however. Lowering prices for healthy foods and menus could be an approach, as family financial restraints and high prices for healthier options at schools may constitute barriers to healthy eating [32].

While different stakeholders developed FLAL [9], this study calls attention to the lack of coordination between governmental institutions and the lack of stakeholder involvement in its implementation, monitoring and evaluation. This may have lead to a demotivation of school staff. A lack of government coordination of school-based obesity prevention programs has also been reported by stakeholders from other countries [32]. In contrast, a study including primary school headteachers in the UK highlighted their wish to be actively involved in public health decision-making [30].

Finally, in Chile and elsewhere, the critical period for becoming overweight in childhood is before the age of 6 years [2, 46]. In fact, although this study did not assess BMI, most children in the participating primary schools were observed to be overweight or obese. Therefore, further preventive interventions might be necessary during the toddler and preschool years.

In summary, to better implement childhood obesity prevention measures like those established by FLAL in schools, policy makers should ensure the involvement of school stakeholders from the beginning. They should promote better coordination between governmental entities which outlasts changes in the ruling party. To transform children’s lifestyles, curricula will have to be revised to mainstream sound nutrition and healthy lifestyle education. These should lead to active student involvement, the acquisition of new skills and the exposure to new foods. A precondition must be the training of teachers, improving their skills to involve students and parents and empowering them to be role models for their students.

### Home environment

The home environment is known to be a key factor in the promotion of a healthy lifestyle, as described in the literature [47]. In qualitative studies, different key stakeholders from the school environment have described the parents’ decisive influence on their child’s lifestyle [30, 32]. The results of the present study revealed a complex interaction between the parents’ lack of problem awareness regarding childhood obesity, lack of time due to high workload and an unhealthy lifestyle within the family. Firstly, most parents were not aware of their children’s unhealthy nutritional status, a phenomenon which has been reported previously for Magallanes and populations in the US and elsewhere (e.g. [47–50]). It is particularly common among low-income families [51]. Unconscious parents make it hard for governments to succeed with any approach.

Secondly, easy access to junk food at home was identified as a further determinant of childhood obesity in Magallanes. This is in line with the literature, which shows that home availability of healthy and unhealthy foods is positively associated with its intake [52, 53]. In a qualitative study with low-income Latino adolescents in the US, parents were described as a barrier to healthier dietary habits through nutrient-dense family meals and the purchase of high-calorie low-nutrients foods [54]. Parents in Portugal on the other hand used reduced availability of certain sugar-containing foods at home as a strategy to limit their children’s intake of these foods. They expressed difficulties in identifying sugar in foods, however [45]. Our results suggest a high intake of SBBs at home, in line with a study carried out 2016 in Santiago, Chile [7]. Chung et al. found that social norms and expectations regarding SBBs were important factors influencing parents’ and grandparents’ decisions to allow their consumption in pre-school children in Australia [55]. As high levels of SBB are consumed in Chile, it might be difficult even for conscious parents to withstand this social norm.

The results of the present study also reveal that parents are not perceived to be good role models for a healthy lifestyle by children and school staff. Parental role modeling was identified by children, adolescents and other stakeholders in schools as key components leading to healthier student diets and increased physical activity, however [31–33, 54]. To what extent our findings are the result of parents’ limited awareness of how their behavior shapes their children’s habits, as Marilia suggests [45], cannot be answered based on our findings.

Our findings further reveal that families consider healthy foods to be too expensive. Since our data were collected, this aspect may have been exacerbated by the pandemic, particularly for mothers with a low socioeconomic status (SES), as FDGs in the capital of Santiago reveal [41]. This underlines the importance for policy makers to also focus on the home environment and on improving the accessibility of nutrient-dense foods.

Thirdly, the present study highlights how parents seem to ignore the impact of high levels of screen time on health or be unable to provide alternatives due to difficult working conditions. Heitzinger et al. [48] showed a correlation between screen time and childhood overweight and obesity in Magallanes, while a systematic review confirmed associations between screen time and greater obesity, higher energy intake, poorer dietary quality and quality of life in children and adolescents [56]. Media consumption further correlates with children’s exposure to commercials [10, 57]. The FLAL, in its first phase, did not restrict TV advertising during all times of the day and only banned ads directed at children based on advertising techniques or the program. Sports-related marketing, shown to lead to unhealthy food consumption patterns in children and youth [58], was therefore still allowed. Moreover, our findings reveal that in the study region, caretakers do not control children’s media access and choice of programs, possibly limiting the effects of the law in this phase to some extent. Alruwaily et al. found, for instance, that child influencers on YouTube mainly promote unhealthy foods [59]. Therefore, limiting screen time in children and having a certain control over the content is a further crucial step in trying to resolve the childhood obesity problematic. In Chile and other countries with strong public health policy measures, this seems not to have been tackled so far. Parental awareness needs to be created, so they serve as role models: By reducing their own screen time, they have a positive influence on their children’s screen-based behavior [60]. At the same time, parents need to have more favorable working conditions, so they have alternatives to leaving their children alone or with inappropriate childcare.

In summary, a higher problem awareness and the importance of setting an example as a parent seem to be key factors that might influence their own behavior regarding nutrition, physical activity and screen-time. Parents themselves are influenced by their community and its environment, however. Interventions aiming at the promotion of a healthy lifestyle should therefore be reframed to involve schools, families and local communities for maximum impact [35, 37]. Healthy foods should be made affordable for all families, especially among vulnerable population groups. Furthermore, in our study region, long working hours present an obstacle to any family or community-based program and need to be considered accordingly.

### Children’s awareness and behavior

We found that children knew the black FoP warning labels, which allowed them to differentiate foods high in sugar, saturated fat and sodium. While Arrua et al. showed that warning signs significantly discouraged Uruguayan children’s choice in both product categories in an experimental design [61], our findings do not suggest that labels influenced children’s food choices in everyday life. This is in line with other studies which showed that children understood different food or menu labelling systems, but that the impact on dietary habits seemed to be limited in real-world studies [62, 63]. In our study, desensitization might play a role. In fact, Chilean mothers expressed a certain label fatigue five years after the implementation of the FLAL [41]. The warning labels seem to be especially important for foods formerly perceived to be “healthy” by children and their parents, however [13, 41, 61, 64].

The approach chosen in Chile, which combines FoPL with nutrition education, had some impact on children’s nutrition knowledge. In line with other studies carried out in Chile, it also made them agents of change in some households, by requesting healthier snacks for instance [13]. In contrast, FoP labels without educational strategies did not influence healthfulness perception of children 6–9 years of age [64]. This underlines the importance of the combination of measures. However, as mentioned above, schools’ nutrition education measures in the study region could be improved in quantity and quality.

In line with this study, literature shows that for primary school children, knowledge does not necessarily translate into healthy behaviors. Aspects like social acceptability, eating context, texture, pleasure and versatility influence their food choices [14], while other studies underline the importance of taste on preschooler and adolescent food selection [65].

Our results reveal the narrow range of foods children in the study region are exposed to at home, possibly partially due to environmental factors like high prices of fresh foods in this remote region. This may lead to the reported rejection of all unfamiliar or unpopular foods at school. There is a vast body of literature underlining how import the familiarity of foods is for child acceptance, and how it is shaped in the first years of life (e.g. [51, 66]). Children in our study region are being exposed to a low dietary diversity, especially regarding fruit and vegetables. They are being conditioned to prefer energy-dense and nutrient poor foods. Therefore, dietary interventions at schools may set on too late. Children need to be exposed to new or disliked foods repeatedly at an early age, by tasting them in a supportive environment [51]. One promising way would be to include experiential learning approaches [67] and hands-on experiences like cooking education [68]. That way they may also be repeatedly exposed to new foods and be more likely to accept them, since they are involved in their preparation.

### Community environment

Regarding leisure time activities, the results of the present study suggest that children consciously opt for screen-based activities, partially because of scarce alternatives. Neighborhood safety issues were barriers to physical activity for children and adolescents mentioned by headteachers, parents and students in other contexts [30, 31]. Additionally, a higher probability of children being active has been reported in favorable community environments (e.g. lower street connectivity, higher safety from crime, walking/cycling facilities, suitable play areas) [57, 69]. In our findings, a further barrier to physical activity imposed by parents was the cold weather. School staff and parents in other contexts, however, opined that playing outside despite the cold weather helps to build self-confidence in children [33]. All these factors supporting physical activity in children are lacking in our study area. In summary, children in our study region are very much influenced by their families, schools and their environment, as elsewhere. The fact that children are left unsupervised for many hours a week is a big problem. For policy makers, considering the establishment of affordable after-school childcare, where different activities for children are offered, would help limit childrens’ screen-time, while enhancing their possibility to be physically and mentally active. Furthermore, it would create another formal structure in which to promote a healthy lifestyle.

### Limitations

We are aware that our research has several limitations. The first is that only schools pertaining to the Health Promoting Educational Establishments participated in the study on a voluntary basis. Their accomplishments had been evaluated very well by the government. Thus, we might have a positive selection bias, not including schools in our sample which face the biggest difficulties in promoting a healthy lifestyle. Moreover, no schools attended by children with a high or a low SES were included in the study, and that the number of formal interviews with school directors is low. A higher number of interviews with representatives from different types of schools might have revealed interesting disparities. The second limitation is that we did not interview the parents of the children, who could have added a “missing piece” regarding the home environment. Moreover, the time span between the implementation of FLAL and data collection may have been too short to identify a notable change in awareness and lifestyle habits. The qualitative design of the study further does not allow for the identification of causal relationships.

This study was carried out in Magallanes, a remote region of the country. Although the results cannot be generalized to other regions in Chile, they may indicate some of the special challenges peripheral regions in Chile face.

## Conclusions

The current study shows that in a peripheral region, the influence of the Chilean Food Labeling and Advertising Law on school programs and children’s lifestyle is limited. Schools are subject to a challenging environment, where multiple demands are placed on them by governmental institutions without their necessary involvement in monitoring and evaluation, empowerment, expert support and funding. Parenting behavior and parental modeling of a healthy lifestyle are lacking, and caretakers hardly get involved in school activities. In conclusion, policies and laws on childhood obesity prevention need to actively involve the key stakeholders like schools in the design, implementation, monitoring and evaluation of these measures. Furthermore, healthy lifestyle approaches must be part of educational curricula, while educators need to be provided with the necessary skills and schools with the necessary funding. Furthermore, strategies must actively engage parents and communities, and must change some of the framework conditions like caregiver work schedules or after-school childcare to be successful.

## Data Availability

The datasets generated and analyzed during the current study are available in the Zenodo repository at the following link: https://zenodo.org/record/6567017.

## Abbreviations

CESFAM: Centros de Salud Familiar (Health Centers)
FLAL: Food Labeling and Advertising Law
FoPL: Front-of-package label
SEREMI: Secretarías Regionales Ministeriales de Salud (regional department of the Chilean Ministry of Health)
SES: Socioeconomic status
SSB: Sugar-sweetened beverages
TEI: Total energy intake
US: United States of America
